# *Aspergillus fumigatus* Challenged by Human Dendritic Cells: Metabolic and Regulatory Pathway Responses Testify a Tight Battle

**DOI:** 10.3389/fcimb.2019.00168

**Published:** 2019-05-22

**Authors:** Mugdha Srivastava, Elena Bencurova, Shishir K. Gupta, Esther Weiss, Jürgen Löffler, Thomas Dandekar

**Affiliations:** ^1^Department of Bioinformatics, Biocenter, University of Würzburg, Würzburg, Germany; ^2^Department of Internal Medicine II, University Hospital of Würzburg, Würzburg, Germany; ^3^EMBL Heidelberg, Structural and Computational Biology, Heidelberg, Germany

**Keywords:** infection, dendritic cells, *Aspergillus fumigalus*, metabolic modelling, signalling

## Abstract

Dendritic cells (DCs) are antigen presenting cells which serve as a passage between the innate and the acquired immunity. Aspergillosis is a major lethal condition in immunocompromised patients caused by the adaptable saprophytic fungus *Aspergillus fumigatus*. The healthy human immune system is capable to ward off *A. fumigatus* infections however immune-deficient patients are highly vulnerable to invasive aspergillosis. *A. fumigatus* can persist during infection due to its ability to survive the immune response of human DCs. Therefore, the study of the metabolism specific to the context of infection may allow us to gain insight into the adaptation strategies of both the pathogen and the immune cells. We established a metabolic model of *A. fumigatus* central metabolism during infection of DCs and calculated the metabolic pathway (elementary modes; EMs). Transcriptome data were used to identify pathways activated when *A. fumigatus* is challenged with DCs. In particular, amino acid metabolic pathways, alternative carbon metabolic pathways and stress regulating enzymes were found to be active. Metabolic flux modeling identified further active enzymes such as alcohol dehydrogenase, inositol oxygenase and GTP cyclohydrolase participating in different stress responses in *A. fumigatus*. These were further validated by qRT-PCR from RNA extracted under these different conditions. For DCs, we outlined the activation of metabolic pathways in response to the confrontation with *A. fumigatus*. We found the fatty acid metabolism plays a crucial role, along with other metabolic changes. The gene expression data and their analysis illuminate additional regulatory pathways activated in the DCs apart from interleukin regulation. In particular, Toll-like receptor signaling, NOD-like receptor signaling and RIG-I-like receptor signaling were active pathways. Moreover, we identified subnetworks and several novel key regulators such as UBC, EGFR, and CUL3 of DCs to be activated in response to *A. fumigatus*. In conclusion, we analyze the metabolic and regulatory responses of *A. fumigatus* and DCs when confronted with each other.

## Introduction

*Aspergillus fumigatus* is an airborne fungal pathogen which can cause a hypersensitive reaction, mucosal colonization, and even life-threatening invasive infection in the immunocompromised host (van de Veerdonk et al., 2017). As the number of intensive care units rises, bone marrow transplantations as well as acute leukemia cases with impaired immunity rise. Virulence traits for this fungus involve non-classical and immune evasion pathways (Amich and Krappmann, 2012). The successful colonization of the fungi depends on the complex interaction of them with human innate and acquired immunity (Cramer et al., 2011; Heinekamp et al., 2015) and determines the successful colonization of the fungi. The inhaled conidia are removed by the cillii of the respiratory epithelium; the smaller conidia avoid this defense and enter the respiratory tract of lungs to be further attacked by alveolar macrophages, dendritic cells (DCs), and other activated leukocytes. If conidia escape they germinate to form hyphae and invade the lung and other organs. The next line of defense is acquired immunity.

Dendritic cells (DCs) serve as a bridge between the innate and the acquired immunity. DCs are antigen presenting cells that express several pattern recognition receptors (PRRs) that recognize *A. fumigatus* and release inflammatory mediators including various cytokines and chemokines to guide other immune cells to the site of infection (Fliesser et al., 2016). DCs internalize both *A. fumigatus* conidia and hyphae and undergo maturation to instruct CD4+ T-cell response to fungi (Stephen-Victor et al., 2017). Protection against regulated immune responses of human DCs is one of the vital strategies for the survival of *A. fumigatus* during infection. The site of infection can be considered as a closed system where the host and pathogen share or compete for nutrition and produce metabolic waste products. Any alteration at this site is sensed by both the host and pathogen and is used to modify the system to its own advantage (Olive and Sassetti, 2016). Several pathways fundamental for the manifestation of the disease have been studied, however, information on the detailed metabolic status of host cells and fungi during infection is still scarce. Moreover, an alarming rise in antimycotic resistant strains (Sanglard, 2016; Choera et al., 2017; Perlin et al., 2017) warrants the identification of new potential targets from *A. fumigatus* metabolism for antimycotic therapies.

We performed metabolic network reconstruction of the central metabolism of *A. fumigatus by* comparing different *Aspergillus* genome sequences and their well-curated metabolic enzyme annotation, followed by a flux balance analysis (Schwarz et al., 2005). This identified those pathways of the central metabolism available for *A. fumigatus* and DCs. Transcriptome data from *A. fumigatus* and DCs infected with *A. fumigatus* (Czakai et al., 2016) was used to quantify the activities of the different pathways (flux strengths of the elementary modes). We studied the metabolic adaptation using three approaches: (i) we looked at the enrichment of pathways according to gene expression data mapping them on the metabolic map and looking which pathways are overrepresented in their enzymes, (ii) we calculated the elementary modes in the overrepresented pathways; (iii) we calculated the flux strength according to the gene expression data.

Different amino acid metabolic pathways and folic acid biosynthesis pathway were active in *A. fumigatus*. Also, we found a higher activity of stress response enzymes such as aldehyde dehydrogenase and catalase. This was further confirmed by qRT-PCR experiments. The DCs show a high activity of fatty acid metabolic pathways. Besides the well-known interleukins, we identified new key regulatory subnetworks active in DCs fighting *A. fumigatus* infection.

## Results and Discussion

Our **Starting Hypothesis** was that the infection environment and the DC challenge is a strong, sometimes deadly challenge for *A. fumigatus*. Nevertheless, specific pathways, for instance regarding energy generation, should be specifically activated. In contrast, DCs as immune cells are adapted and optimized for fighting infection including fungi. This led to the hypothesis that specific pathways for fighting *A. fumigatus* infection such as redox pathways should be mainly activated. As we go over the analysis flow and the detailed results we can see that both hypotheses were step by step replaced by novel insights on a number of specific metabolic responses in pathogen and host and these in turn were mediated by regulatory changes for which again several key players could be identified. Finally, we validated these partly unexpected results by a 2nd experimental data set (in supplement) using RT-PCR measurements on the key enzymes identified.

### Analysis Flow

**Figure 1:**
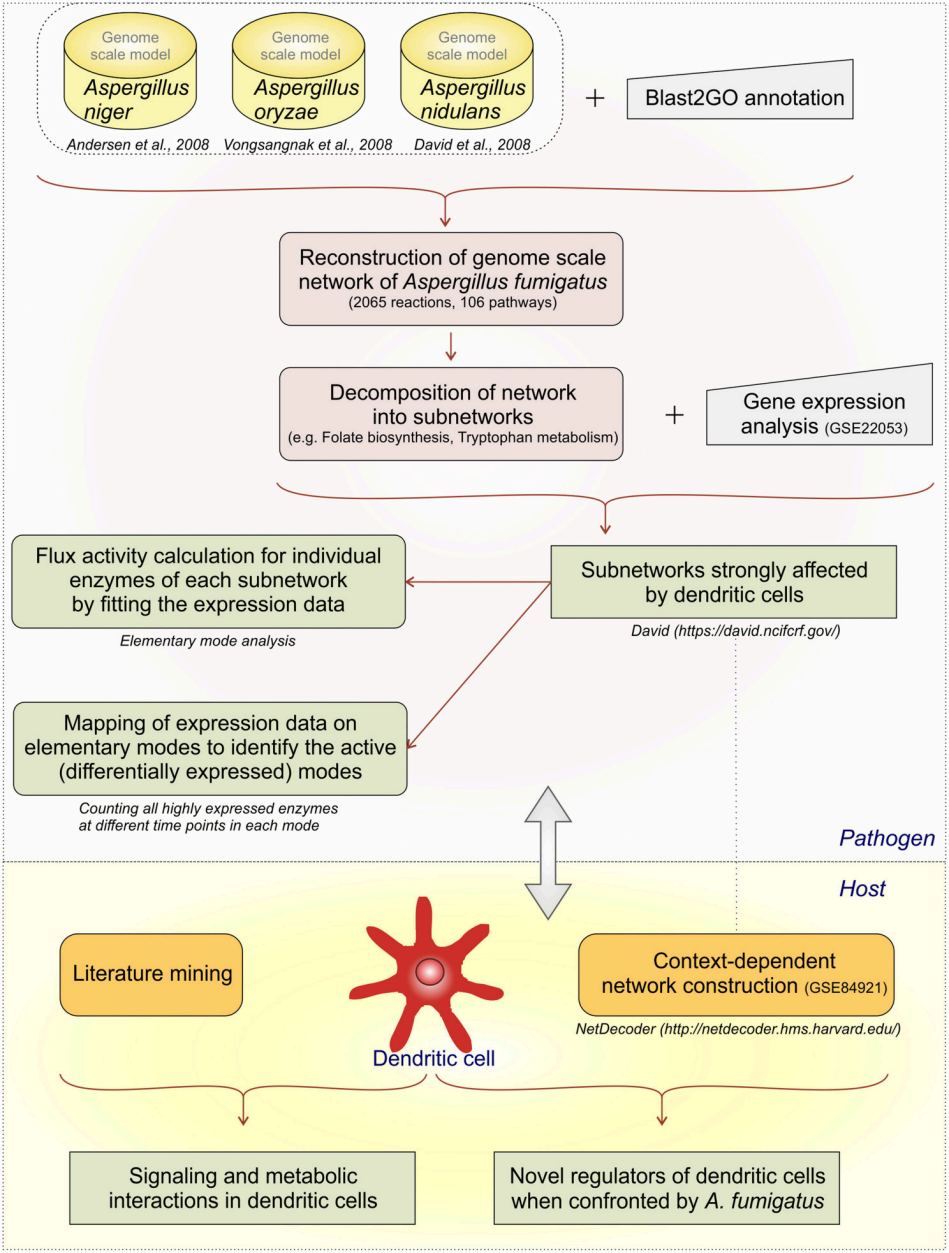
Analysis flow. The different analysis steps on *A.fumigatus* and the host response are given.

We develop first a genome-scale model and then stepwise reduce it to subnetworks using both metabolic flux modeling and enrichment analysis on the subnetworks (Figure 1):

1. We created a large network as an initial genome-scale network which includes not only the reactions from the central metabolism but also reactions from propanoate metabolism, seleno-compound metabolism, terpenoid biosynthesis, and others.
2. However, calculation of all EFMs (elementary flux modes) becomes computational challenging for such a huge network, there is combinatorial explosion. The initial network was decomposed into subnetworks (Schuster et al., 2002; Schwartz et al., 2007) considering individual parts of the metabolic map individually.
3. Next we mapped the gene expression data to the subnetworks. By this we identified those subnetworks which responded strongly to the challenge to be confronted with DCs. Specifically, these were the metabolic subnetworks for ascorbate and aldarate, beta-alanine, fatty acid degradation, glycolysis/gluconeogenesis, folate biosynthesis, arginine and proline, tryptophan as well as degradation of valine, leucine, isoleucine.
4. In those subnetworks which were affected we calculated the elementary modes (Supplementary Table S2). Seventy-nine flux modes were calculated in the affected eight subnetworks.
  We did do enrichment analysis mapping the gene expression data on the elementary modes. We counted how often one enzyme occurs in elementary modes. Including this step in our analysis path we were able to predict the important elementary modes and enzymes during infection conditions. For instance, in the subnetwork ascorbate and aladarate metabolism, the enzyme inositol oxygenase converting myo-Inositol to D-Glucuronate was found to be upregulated at all-time points (6, 9, and 12 h).
5. We then calculated flux strengths for individual enzymes (Supplementary Figure S1). Apart from the enzymes observed in step 4 we were able to identify other important enzyme for instance potassium activated aldehyde dehydrogenase which showed strong flux both in arginine and proline metabolism and beta-alanine metabolism. Further important enzymes and their activities are discussed below.
6. For the dendritic cell expression data from the dendritic cell infected by *A. fumigatus* (GSE69723) was used to create a context dependent network. Mining information from publications was useful to create links between the metabolic and regulatory pathways. NetDecoder was used to identify the key regulators in DCs during infection.
7. Finally, we re-checked several of the expression predictions by independent RT-PCR experiments and collected these data. Such a second validation by experiments is typical in modeling studies, for instance (Cecil et al., 2015) use metabolite measurements and gene expression data, and (Schwarz et al., 2007) use independent gene expression data-sets for verification of their bioinformatics models.

### Genome-Scale Metabolic Model

The *A. fumigatus* metabolic network created used the orthology information from the available metabolic models for the three other well-known *Aspergillus* fungi, *Aspergillus niger* (Andersen et al., 2008)*, Aspergillus nidulans* (David et al., 2008) and *Aspergillus oryzae* (Vongsangnak et al., 2008). Further, the Kyoto Encyclopedia of genes and genomes (KEGG) database (Kanehisa and Goto, 2000) was used to fill in the missing information and gaps. Simulation based methods are needed to characterize the functional relationships in the model as the network is too complex and numerous to analyze manually.

The resulting network content includes 2,065 reactions and 106 metabolic pathways (Supplementary Table S1). Pathways included the carbohydrate metabolism, amino acid biosynthesis and degradation, fatty acid metabolism, and nucleotide biosynthesis. Reactions from secondary metabolism such as the metabolism of cofactors and vitamins, the metabolism of terpenoids and polyketides, as well as the lipid metabolism were also included in the network.

In a metabolic network, EFMs are defined, minimal sets of enzymes that support steady-state operation of a metabolic network. Irreversible reactions have to progress in their unique direction (Schuster et al., 1999, 2000, 2002). However, the computation of EFMs in genome-wide models of metabolism is challenging as there is usually a combinatorial “explosion” of enzyme combinations, a very high number of modes difficult to calculate results. Hence, the individual metabolic maps as annotated from our genome scale model are considered as subnetworks. These subnetworks are not the textbook pathways instead they are specific to *A. fumigatus* and depend on the set of enzymes available. The *A. fumigatus* enzymes were included and identified according to orthology analysis from the other fungal species and Blast2Go annotation as discussed before.

### Elementary Flux Mode Calculation in Subnetworks

**Figure 2:**
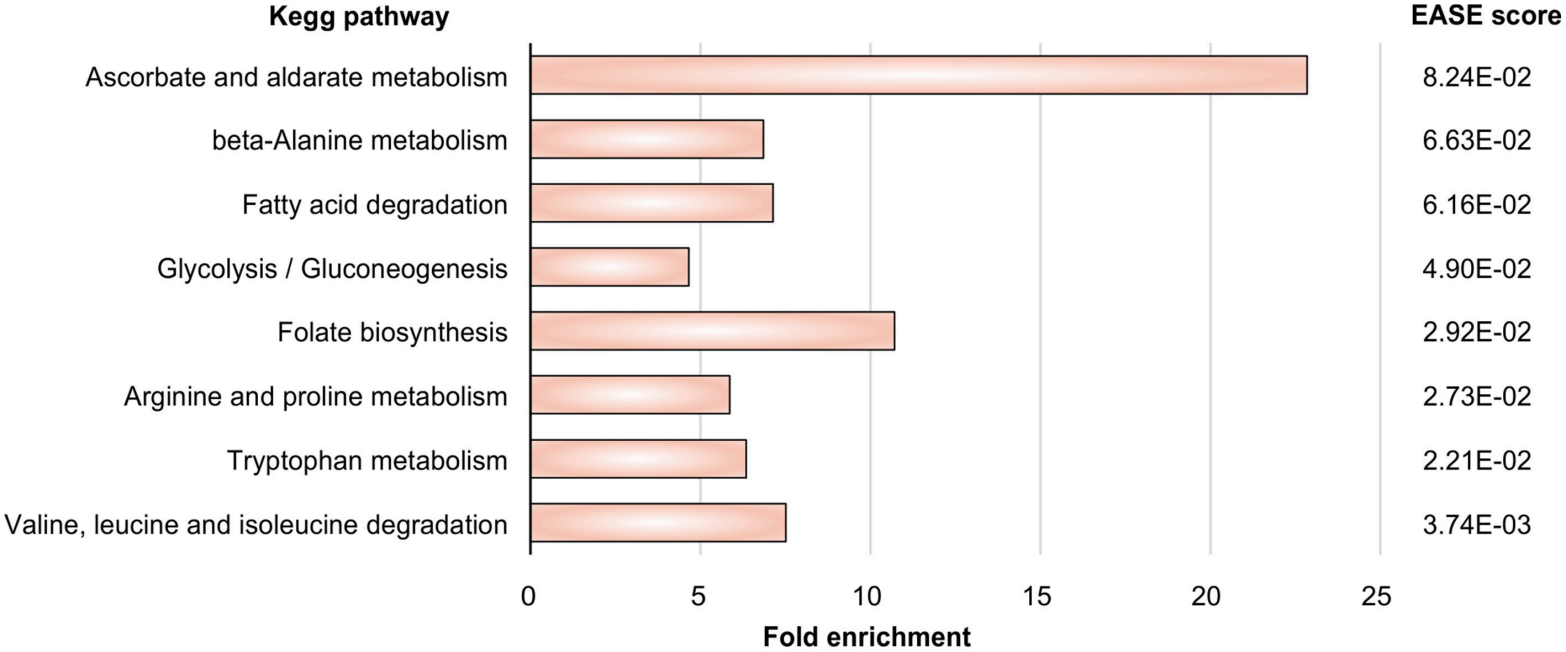
Pathway enrichment. Metabolic pathways in *A. fumigatus* significantly enriched during infection in human dendritic cells.

Gene expression signatures (data set GSE84921) revealed alterations in pathogen metabolic pathways contributing to circumvention of host immune mechanisms. Our approach (Figure 1) tests first which metabolic subnetworks change in *A. fumigatus* under DC challenge (Rezola et al., 2013): we count for which subnetworks the number of overexpressed enzymes is significantly higher in the DC challenge condition compared to other pathways and normalizing for pathway lengths using DAVID. Eight metabolic subnetworks were observed to be significantly overrepresented in *A. fumigatus* during infection of DCs according to EASE score (Figure 2; significance threshold ≤ 0.1). Since we aimed to focus only on the pathways which were overrepresented in *A. fumigatus* when challenged with DCs, we then looked in detail at the pathways around these eight subnetworks. Supplementary Table S2 gives all the elementary modes calculated for all the enriched metabolic pathways in *A. fumigatus* and Supplementary Table S3 details the different stoichometric matrices for all the enriched metabolic pathways in *A. fumigatus* during infection.

All the subnetworks which actually run for metabolic flux calculation are given in YANAsquare compatible dat files (Supplementary Dataset 1). The big genome-scale metabolic model is not sufficiently amenable (too big) nor sufficiently curated to allow direct transfer to a software calculation and so we simply provide there all reactions considered and alert the user about this limitation.

EFMs characterize the optimal route of utilizing external substrate and forming external products considering the metabolism of the whole cell (Chinnasamy Perumal et al., 2014). Here, 159 flux modes were computed, out of which 79 EFMs were significantly up or downregulated upon encounter of human DCs. These EFM_S_ involved 13 reversible and 66 irreversible reactions (Supplementary Table S2). The stoichiometric matrix for each subnetwork is also detailed in Supplementary Table S3. Gene expression and mapping to KEGG pathways is a first indication on all metabolic changes. However, Elementary Mode Analysis (EMA) calculates all possible pathways accessible for a set of enzymes. Therefore, the advantage of EMA compared to looking up the pathways in KEGG or in a text book is to identify all balanced pathways for a given set of enzymes. This includes all mixtures between text book pathways which are balanced and then may provide very useful combinations of metabolites in high yield for an organism. For example, for glycolysis coupled to pentose phosphate pathway this includes mixtures providing ribose sugars for nucleotide synthesis or high yield of NADPH for synthesis as well as special pathways such as futile cycles to create heat or highly sensitive metabolic responses. This higher resolved picture for the enzymes in *A. fumigatus* using EMA becomes even more detailed considering different flux strength for the individual pathways, for instance larger investment in energy generation, generation of reduction equivalents or nucleotide synthesis. Hence, every subnetwork identified by the gene expression comparison was subsequently modeled by EMA and flux strength calculation to achieve such a better resolved picture of the metabolic change.

The fluxes calculated by YANAsquare are only relative activities. This has the advantage that these can be easily calculated from further available data, even gene expression data. However, unless you normalize the measurement by metabolite concentrations, you get only a relative estimate. Furthermore, the strongest flux in such a comparison is always 100% per definition and not by actual measurement. In a weakly active system the percentages may be misinterpreted. However, we focus specifically on *A. fumigatus* and dendritic cell subnetworks with marker pathways that are highly active in specific subnetworks upon confrontation.

### Mapping of Gene Expression Data on Elementary Modes

**Figure 3:**
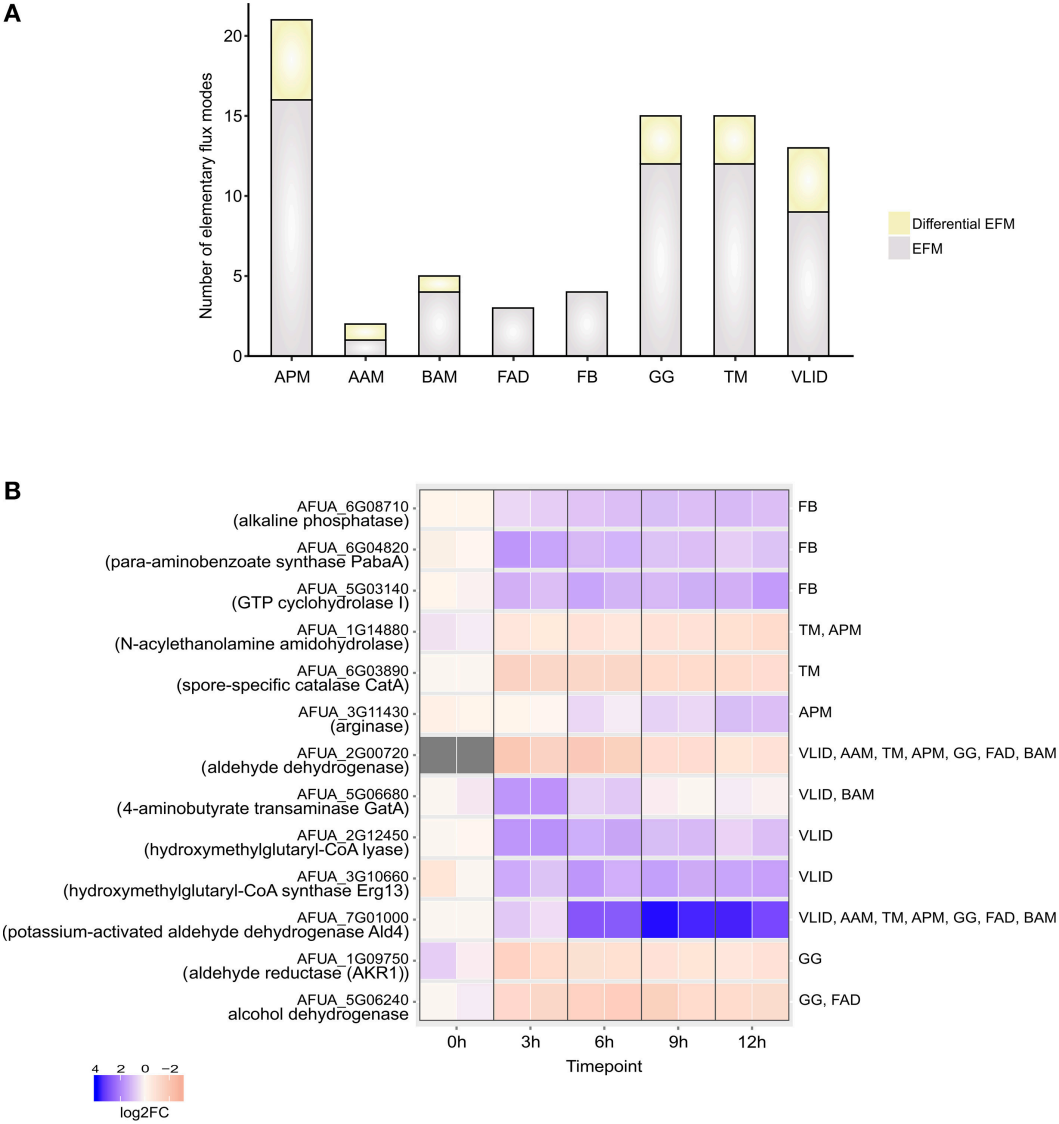
Elementary Flux modes. **(A)** Total number of EFMs (gray + yellow) and differential EFMs (yellow) computed in A. fumigatus metabolic pathways significantly enriched during infection in human dendritic cells. **(B)** Expression of enzymes involved in differential EFM at different time points. APM, Arginine and proline metabolism; AAM, Ascorbate and aldarate metabolism; BAM- beta-Alanine metabolism; FAD, Fatty acid degradation; FB, Folate biosynthesis; GG, Glycolysis / Gluconeogenesis; TM, Tryptophan metabolism; VLID, Valine, leucine and isoleucine degradation.

The EFMs were classified into EFMs that changed under DC challenge in *A. fumigatus* (Figure 3A) on the basis of gene expression data

In the ascorbate and aldarate metabolic pathway, differential EFM carry the fluxes through highly expressed myo-inositol oxygenase enzyme which transforms inositol into glucuronic acid using oxygen. The following reaction is catalyzed

Myo-inositol + Oxygen –> Glucuronic acid + H_2_O

Glucuronic acid has several essential functions in cellular metabolism (Kuivanen et al., 2016). It can be used as a precursor for UDP-glucuronic acid and can also serve as an energy source for growth (Moyrand and Janbon, 2004). However, the presence of UDP-glucose dehydrogenase (Ugd1) suggests the presence of two different pathways for the biosynthesis of UDP-glucuronic acid in *A. fumigatus*. This has been established for yeast and plants. Capsule biosynthesis that is strictly required for virulence and growth at 37°C was reduced in ugd1Δ strains of a pathogenic fungus *Cryptococcus neoformans* (Bar-Peled et al., 2004; Moyrand and Janbon, 2004). However, the role of Ugd has not been well-characterized for *A. fumigatus* with the assumption that it may have a role in host immune system activation (Fontaine et al., 2009). The EMA and flux activity calculation from our model also suggest the important role of glucuronic acid for immune activation and host cell adaptation. The beta-alanine metabolic pathway was also enriched in *A. fumigatus* during infection conditions. The reactions active in beta-alanine metabolism are the synthesis of spermine via the utilization of spermidine, acetyl-coA, and propenoyl-coA which has been implied for fatty acid biosynthesis. Four genes for folate biosynthesis have been enriched during infection in DCs. It has been previously demonstrated that folate biosynthesis is an important pathway and mutants of folate biosynthesis pathways are avirulent in *A. fumigatus*. Folate biosynthesis is required not only for infection and germination but also for maintenance (Schoberle and May, 2007; Dagenais and Keller, 2009). The assimilation of nitrogen is crucial for fungi to establish a successful infection and ensure their survival. *A. fumigatus* can convert nitrate into ammonia and can also utilize the different amino acids. Amino acids are substrates of protein translation and hence are necessary for growth glutamine and arginine is the preferred nitrogen source for *A. fumigatus* during nitrogen starvation (Krappmann and Braus, 2005). The arginine pathway is also enriched during infection in dendritic cell. As valine, leucine, and isoleucine are the essential amino acids for *A. fumigatus* which are not readily available during infection, *A. fumigatus* utilizes them by degradation of valine, leucine, and isoleucine from the host (Vödisch et al., 2011; Oliver et al., 2012). Moreover, there is an *A. fumigatus* isoleucine/valine auxotroph. The enzyme dihydroxyacid dehydratase (ilv3A) is lacking (Oliver et al., 2012). It is avirulent in systemic infection. Tryptophan is also an essential amino acid which is not available in the human host environment and the fungus has to produce independently. Auxotrophs of aromatic amino acids have been observed to have attenuated virulence in neutropenic mice (Sasse et al., 2016). The tryptophan pathway was highly upregulated in *A. fumigatus* during dendritic cell infection conditions as estimated by our model. Tryptophan biosynthesis has been suggested as an excellent pharmacological target in *C. neoformans* (Fernandes et al., 2015) and our results suggest that they can be an interesting therapeutic target in *A. fumigatus* as well. However, this requires detailed investigation along with auxotrophic studies (Schoberle and May, 2007). The results from our enrichment analysis show that *A. fumigatus* has an active fatty acid metabolism which ensures that the fungus is capable of using alternative carbon sources in the stressful host environment (Olivas et al., 2008).

**Figure 4:**
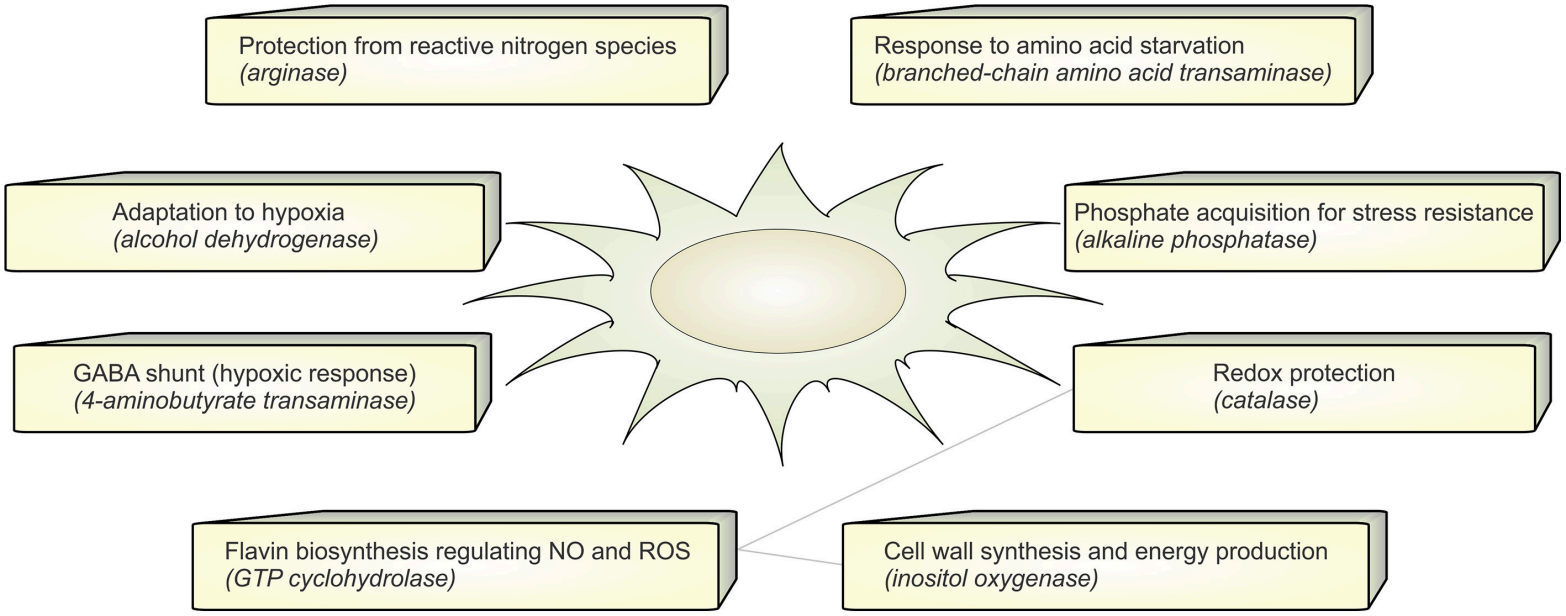
Functional annotation. The figure summarizes the active metabolic enzymes in *A. fumigatus* during interaction with dendritic cells. Each enzyme is associated with their possible biological function in order to adapt to stressful environment in dendritic cells.

In the set of calculated EFMs; the occurrence of the enzymes associated with EFMs that change flux under infection reflects the importance of that enzyme in *A. fumigatus* during infection. We identified that aldehyde dehydrogenase is involved in 5 differentially regulated EFMs. It is evident that the evolutionarily conserved aldehyde dehydrogenase has a crucial function by providing tolerance to the organism against a variety of stresses, including oxidative stress (Singh et al., 2013). The top-ranking differential EFM enzymes (Figure 3B) were validated using qRT-PCR experiments (see below). The metabolic response in *A. fumigatus* during interaction with DCs is summarized in Figure 4 and Supplementary Table S4.

### Flux Activity Changes

To determine which of the pathways are active during the infection conditions YANAsquare calculates the activities of individual enzymes according to the experimental data. Evolutionary algorithm routines in YANAsquare allows the inclusion of fitness parameters and calculates best possible solutions for enzyme fluxes on the basis of experimental expression data (Schwarz et al., 2005). However, sufficient data points are needed to even out errors from individual measurements of estimates for flux strengths (Dandekar et al., 2014). In particular, gene expression data only indicate the changes in expression of the enzyme mRNA. Translational regulation, enzyme activity and protein stability are further factors to consider and this yields a controversial and complex picture (see e.g., systematic evaluation and limitations in Machado and Herrgård, 2014). On the other hand, in linear pathways, enzymes work together. This enzyme interaction limits the amount of activity variation. Similarly, branch points and connection hubs such as central enzyme activities constrain the range of possible enzyme activities for the network further. However, using our network approach and estimate this nevertheless leads to an evening out of individual errors as soon as moderate-sized networks of 20–30 enzymes are considered. This was carefully validated by directly measuring metabolite concentrations in another infection model. *Staphylococcus aureus*. The residual error was around 5–10% for the individual metabolite concentrations measured (Cecil et al., 2011, 2015).

The gene expression data-set (GSE22053) we analyzed hence enables a view on the time course of changes at 3, 6, 9, and 12 h (see Materials and methods) when *A. fumigatus* experiences the DC challenges. There is a surprising plasticity of the different pathways involved. Regarding the glycolytic pathway the enzymes lactate dehydrogenase (LDHA), aldehyde reductase and alcohol dehydrogenase demonstrate upregulation of enzyme activity ranging from 30 to 50 percent. This indicates that fungal cells survive in anaerobic conditions in DCs. The activity for alcohol dehydrogenase increases from 17% at 3 h to 33% at 6 h, 40% at 9 h and 43% at 12 h, respectively. During the course of the DC challenge, the down regulated activity for pyruvate kinase and the upregulated activities of phosphoenolpyruvate carboxykinase (PEP carboxykinase) and pyruvate decarboxylase (pdcA) indicates that the fungal cells suffer starvation and hence enter the gluconeogenesis cycle where phosphoenol pyruvate is converted to glucose instead of pyruvate.

The activity of inositol oxygenase in ascorbate and aldarate sugar acid metabolic pathway was found to be very high, close to the maximum activity of flux strength (98% as calculated by YANAsquare) at 3 h, and reaching maximum activity at 6 h. This activity was reduced slightly to 99% at 9 and 12 h. Acyl CoA dehydrogenase was also highly active with the activity values ranging from 80 to 85% at different time points. In the pathway valine-leucine-isoleucine degradation the activity for the enzyme branched amino acid transferase was decreased at all the time points with the lowest activity of 40% at 3 h. The enymes catalase and aldehyde dehydrogenase were also significantly positively regulated with 66 and 69% activity for aldehyde dehydrogenase at 6 and 9 h, respectively.

Aldehyde dehydrogenases (ALDHs) are known to metabolize endogenous and exogenous aldehydes and thus mitigate oxidative stress in both prokaryotic and eukaryotic organisms (Singh et al., 2013). The high activity of ALDHs and catalase indicate oxidative stress in *A. fumigatus* during infection in the dendritic cell. During the pathway enrichment analysis based on the gene expression data set (GSE69723), we did, however, not notice any significant change in other redox pathways.

**Figure 5:**
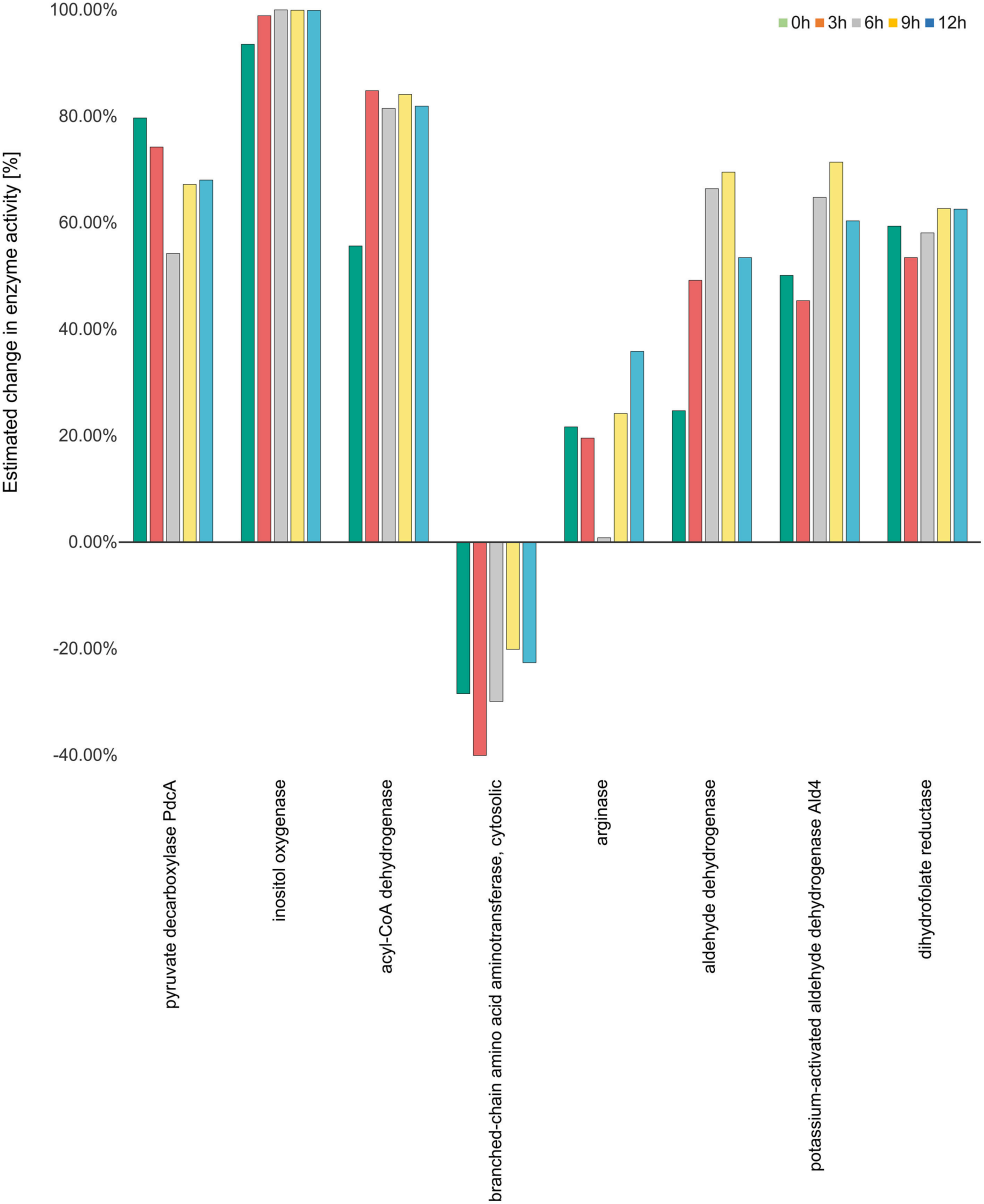
Enzyme activity estimation. Enzyme activity for all the enzymes were calculated by fitting the expression data to the metabolic model. The enzyme activity for inositol oxygenase was observed to be the highest at all the time points, while it was observed to be downregulated for the enzyme branch-chain amino acid aminotransferase. All the other enzymes were observed to be more than 20 percent active at all the four time points.

The activities of enzymes with maximum modulation in activity in all considered pathways are depicted in Figure 5 while the activities of the enzymes for all the over-represented metabolic pathways calculated on transcriptome data (Morton et al., 2011) can be viewed in Supplementary Figure S1.

### Metabolic Changes in Human Dendritic Cell When Infected by *A. fumigatus*

**Figure 6:**
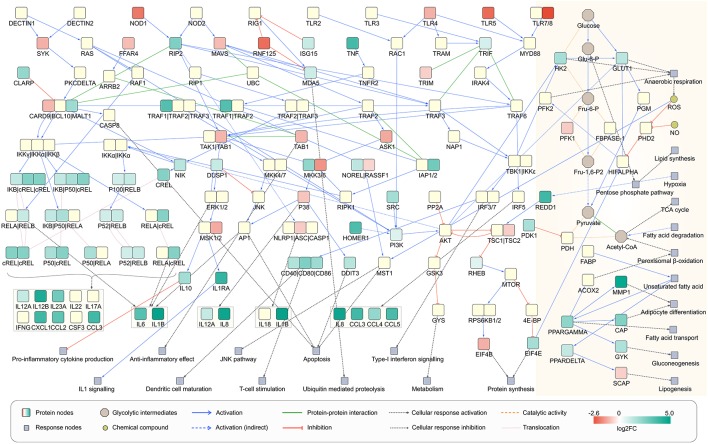
Network view of infected dendritic cells. Dendritic cell response on *A. fumigatus* infection at 6 h. The enzymes involved in the metabolic responses are boxed in yellow (shaded floral white color). The unboxed proteins represent the signaling events that take place during the infection of *A. fumigatus* in dendritic cells.

To understand the metabolic changes in dendritic cell during the infection conditions we used gene expression data set GSE6972 (DCs after and before *Aspergillus* challenge) and analyzed which enzymes are strongly changing under the challenge. To compare by this the affected metabolic or regulatory pathways and how far the challenge modified more regulation or metabolism in DCs we considered links between the affected enzymes according to literature (Pearce and Everts, 2015). The pathways which were affected in the human DCs were hence mapped using the over-representation analysis and gene expression data (Figure 6). Recent studies define the signaling pathways in DCs affected during *A. fumigatus* infection (Czakai et al., 2016, 2017). Here we extend the expression information to map all pathways which were highly expressed during infection.

This resulted in the significant over-representation of five metabolic pathways for >2 fold and several signaling and regulatory pathways (Supplementary Table S5). The over-represented metabolic pathways include fatty acid metabolism, fatty acid degradation, one carbon pool by folate, glycosaminoglycan biosynthesis and valine, leucine, and isoleucine degradation.

The pathogen metabolic pathway over-representation and flux changes indicated the involvement of amino acid metabolism in host infection. For instance, DCs utilize the tryptophan metabolic pathway to increase their fitness and strength against fungal infection (Romani, 2004). Common pathways allow the adaptive immune system to switch on circuits active under amino acid deprivation and/or byproducts furnished from amino acid metabolism (Grohmann and Bronte, 2010). Fatty acid synthesis is involved in the activation of DCs after TLR-induction, the acetate donor for acetylation of proteins was found to be ~2.3 fold upregulated in our analysis (Pearce and Everts, 2015).

Cell surface proteoglycans are composed of a protein core covalently linked with glycosaminoglycans (GAGs). Among the proteoglycans, chondroitin sulfate and heparan sulfate has been reported as modulators of the innate immune response (Wegrowski et al., 2006). At inflammatory sites, GAGs structure and location are altered, which serves to modify the activity of the GAG-dependent soluble and cell surface effectors of the inflammatory process (den Dekker et al., 2008).

The metabolic pathway enriched in DCs indicates that these cells acquire carbon and nitrogen through fatty acid and amino acid sources. On the other hand, the dendritic cell modulates immune functions by upregulating of GAG pathways and DNA synthesis and repair by modulating the one carbon pool pathway via folate.

Regarding concomitant signaling and regulation of the activation of DCs can be induced by a various receptors for pathogen-associated molecular patterns (PAMPs) as well as alarmins. By these signals, DCs become active in infection or to cope with other environmental challenges (Pearce and Pearce, 2013). During the interaction with *A. fumigatus* DCs expressed maturation markers and interleukins but in particular also several C-type lectin receptors, toll-like receptor (TLRs) signaling pathway, RIG-I-like receptors, and Nucleotide-binding Oligomerization Domain (NOD)-like receptors as depicted in Figure 6. Beside C-type lectin receptors pathway all other above mentioned pathways were significantly affected during infection as revealed by over-representation analysis of differentially expressed genes (DEGs) (Supplementary Table S6). The activated signaling cascades stimulate the production of various cytokines and chemokines.

From a metabolic perspective, resting DCs oxidize glucose, trigger oxidative phosphorylation and thus produce lactate. Nevertheless, after environmental contact (e.g., with pathogens) glycolysis and anerobic (Warburg) metabolism becomes more important (Krawczyk et al., 2010). Under this hypoxic condition, pyruvate is not used in the TCA cycle but is instead turned into lactate. To answer the question how the Warburg metabolism is activated in *A. fumigatus* infected cell we made an overlay of the gene expression data on the reconstructed signaling cascade and attempted to connect the cascade with metabolism of infection induced DCs (Figure 6). In cells that have been activated for >6 h, expression of many genes in the DCs. This triggers new functions as reflected by significant enrichment of many signaling and few metabolic pathways (Supplementary Table S5). DCs co-initiate inflammation and prime T-cell responses (Banchereau et al., 2000). The analysis indicated the enrichment of T cell receptor signaling pathways in infected DCs, confirming previous observations (Banchereau et al., 2000) (Figure 6). The significant enrichment of pathogen inducing Toll and NOD like receptor signaling pathway was also observed which further stimulates the NF-kappa B signaling pathway mainly through MAPK cascade and other immune responses (Figure 6). Moreover, cells activated by PAMPs undergo profound metabolism changes which are required for biosynthesis and energy production (Pearce and Everts, 2015). In the following section, we will discuss the metabolic behavior of infected DCs solely based on gene expression data at 6 h of infection.

Nitric oxide synthase iNOS generates nitric oxide (NO) to inhibit the mitochondrial respiration. In macrophages activated by LPS and IFN-gamma or bacterial infection, extracellular arginine is imported into the cell and it is metabolized to NO and citrulline by iNOS as in *Helicobacter pylori* induces macrophages (Gobert et al., 2001; Kelly and O'Neill, 2015). In our EMA we did not find a significant increase in the flux to produce arginine. Based on our observation that there are no significant changes in iNOS expression during the infection, we can discard the possibility of an iNOS mediated activation of Warburg-like glycolysis metabolism in *A. fumigatus* infected DCs.

However, the hypoxia-inducible factor 1-alpha (Hif-1α) transcription factor facilitates the metabolic switch toward anaerobic glycolysis by targeting hypoxia response element containing genes (Semenza et al., 1991; Mole et al., 2009) such as Glucose transporter 1 (GLUT1) and glycolytic enzymes (Chen et al., 2001). The upregulation of the dominant glucose transporter GLUT1 indicates the enhanced uptake of glucose in *A. fumigatus* challenged DCs. Additionally, the increased expression of hexokinase 2 (HK2) that phosphorylate glucose to produce glucose-6-phosphate (G6P) mediates an increased rate of glycolysis in DCs against *A. fumigatus* challenge. LDHA is induced by Hif-1α and hence pyruvate is turned into lactate, and far less ends up in acetyl-CoA for the TCA cycle. The expression of LDHA was significantly enhanced to ~1.5 fold at 6 h of infection. Moreover, Hif-1α activates pyruvate dehydrogenase kinase (PDK) that inhibits pyruvate dehydrogenase, which consequently inhibits the pyruvate decarboxylation. This inhibits the use of acetyl-CoA in the citric acid cycle and cellular respiration by interfering with the transformation of pyruvate into acetyl-CoA. The expression of PDK was significantly increased to >3 fold while no significant change in expression of Hif-1α was detected at 6 h.

### Regulation of Metabolism

There is increased hypoxia-inducible factor 1-alpha inhibitor expression (HIF1AN, ~1.5 fold upregulated). Hypoxic conditions induce expression of egl-9 family hypoxia inducible factor 1 (EGLN1) and factor 3 (EGLN3). Under re-oxygenation they terminate the HIF response rapidly by regulating the stability of HIF-α subunits (Stiehl et al., 2006; Yasumoto et al., 2009). In the complex regulation of Hif-1α, another regulator siah E3 ubiquitin protein ligase 2 (SIAH2) controls various substrates involved in stress and hypoxia responses in their stability, including factor 3 (EGLN3) (Nakayama et al., 2004). The expression EGLN3 was significantly upregulated by >3.5 fold and that of SIAH2 by >1.5 fold in 6 h after infection.

mTOR and its upstream activators PI3K-Akt also serve as regulators of metabolic switch due to their ability to glycolysis and anabolic metabolism (Lewis et al., 2011; Everts et al., 2012; Finlay et al., 2012). Akt can also enhance the glycolytic flux directly by promoting the translocation of glucose transporters to the cell membrane and/or by enhancing the enzymatic activity of the glycolytic enzyme 6-phosphofructo-2-kinase/fructose-2,6-biphosphatase (PFKFB1) by promoting the activity of 6-phosphofructo-2-kinase/fructose-2,6-biphosphatase 2 (PFKFB2), its allosteric activator (Pegoraro et al., 2013). Fructose 2, 6-bisphosphate (Fru-2, 6-P2) is the most potent stimulator of PFKFB1, a key enzyme of glycolysis. Beside HIF-α, mTOR affects the expression of SREBP that controls the expression of genes involved in lipogenesis. PI3K-Akt regulated 6-phosphofructo-2-kinase/fructose-2, and 6-biphosphatase 3 (PFKFB3) expressions also involves in the induction of glycolysis. The expression of Pi3K and PFKFB3 was upregulated while Akt (<1.5 fold) and PFKFB2 were downregulated at 6 h of infection. Knockdown or inhibition of PFKFB3 results in autophagy induction (Klarer et al., 2014). FKFB3 is not only a critical control point in the regulation of glycolytic flux and autophagy, it also mediates biosynthesis of lipids. The peroxisome and fatty acid degradation metabolic pathways showed >2-fold significant enrichment of PFKB3 (Supplementary Table S5), implying fatty acid catabolization by β-oxidation. The role of the peroxisome in the breakdown of very long chain fatty acids through β-oxidation is well-known (Poirier et al., 2006). We also found significant upregulation of the peroxisome proliferator-activated receptor (PPAR) subnetwork. Fatty acids are the most common intracellular ligands for all nuclear receptors of the PPAR family (Chinetti et al., 2000). These often heterodimerize with retinoid X receptor (RXR) and control with transcriptional coactivators fatty acid metabolism (Nagy et al., 2012). PPARα regulates ß-oxidation, PPARβ/δ lipid oxidation and cell proliferation. Transcription factor PPARγ, promotes fatty acid synthesis and adipocyte differentiation and enhances blood glucose uptake. During infection RXRB and PPARα were downregulated while the PPARδ and PPARγ were upregulated. The enriched HIF-1, PI3K-Akt, and mTOR signaling pathways indicate the metabolic behavior of *A. fumigatus* infected DCs somewhat mimics cancer cell metabolism which is notorious for its metabolic shift (Mitchell and Gubler, 1987; Liberti and Locasale, 2016).

Host-derived matrix metalloproteinases (MMPs) are necessary for the successful eradication of infection (Coppens, 1991). MMPs involved in multiple functions including the influx of effector cells, intruder killing, resolution of inflammation, cell proliferation, and remodeling of the extracellular matrix (ECM). We observed the expression changes in five members of MMP family, collagenase MMP1, gelatinase MMP9, stromelysin MMP10, metalloelastase MMP12, and stromelysin MMP19. These all were significantly upregulated at 6 h of infection. The excessive inflammation following infection may cause tissue damage and MMPs are implicated in causing this immunopathology (Coppens, 1991).

Taken together, the complex interplay between several genes regulate the energy requirement of the *A. fumigatus* infected DCs at 6 h by inducing both the metabolic shift toward glycolysis and energy production by metabolism of fatty acid.

**Figure 7:**
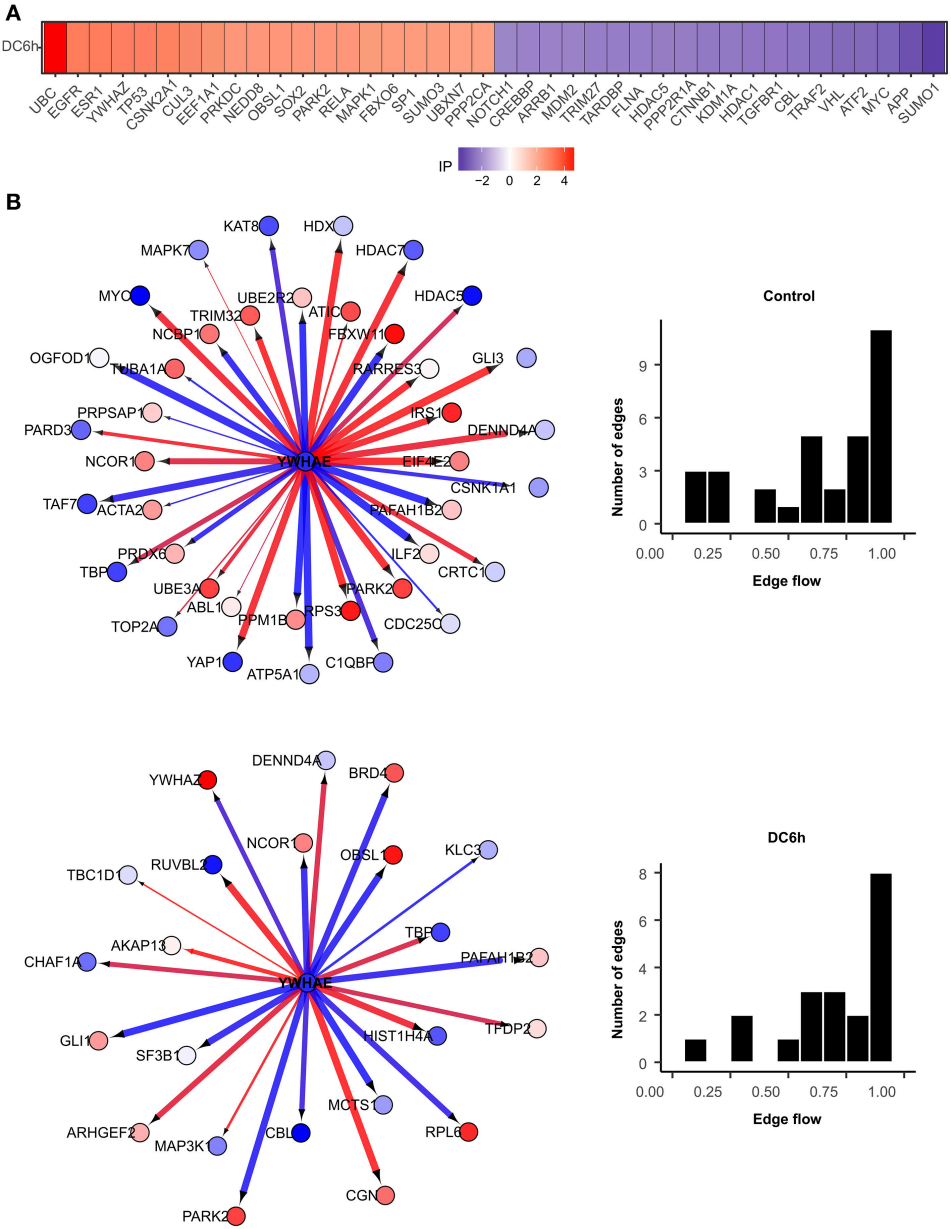
High impact genes. Heat maps for top 40 genes with high impact scores in **(A)** *A. fumigatus* confronted DCs at 6 h. The impact score (IP) is defined in methods. The more extreme the IP score (positive or negative) the more likely it is a gene that contributes to the fungal confrontation. **(B)** Context-specific profiles of confronted key gene motifs. YWHAE acts as one of the network regulatorgenes in confronted DCs. Color of gene nodes is according to node flow differences comparing control vs. confronted (see gradient bar, top). Edge flow strengths is represented by thickness, its direction path connects the source with the target nodes. Red edges indicate a positive gene expression correlation between a pair of protein interactions, while blue edges represent a negative correlation. The edge flow distribution for each gene is shown in the bar chart at right.

We further identified novel genes that play critical roles under during dendritic cell infection to *A. fumigatus* using NetDecoder program (da Rocha et al., 2016) that integrates both PPI network-based and reverse engineering-based approaches via a process-guided flow algorithm for detecting the key regulators. The identified high impact genes are shown in Figure 7A.

Telomerase expression (hTERT) is usually very low and transient in somatic cells (Horikawa and Barrett, 2003) as they are not immortal (Frolkis et al., 2003). A total of 50% of genes regulate hTERT transcriptional regulation according to BioCarta database (Nishimura, 2001) were identified as routers or target genes (Table 1). This indicates the modulation of telomerase activity during DCs infection of *A. fumigatus*. These changes have also been observed in DCs on stimulation with microbial components (Ping et al., 2003).

**Table 1:** Top 40 high impact genes in DCs identified by network analysis.

**Gene symbol**	**Protein names**	**Impact_value**	**Key target**	**Network router**
UBC	Polyubiquitin-C	4.683650567		Yes
EGFR	Epidermal growth factor receptor	3.094751009		Yes
ESR1	Estrogen receptor	3.094013103	Yes	
YWHAZ	14-3-3 protein zeta/delta	2.995119256	Yes	
TP53	Cellular tumor antigen p53	2.983663328	Yes	
CSNK2A1	Casein kinase II subunit alpha	2.929300326	Yes	
CUL3	Cullin-3	2.767055252		Yes
EEF1A1	Elongation factor 1-alpha 1	2.651397012	Yes	
PRKDC	DNA-dependent protein kinase catalytic subunit	2.520674964	Yes	
NEDD8	Neural precursor cell expressed developmentally down-regulated protein 8	2.507748799		Yes
OBSL1	Obscurin-like protein 1	2.506763934		Yes
SOX2	Transcription factor SOX-2	2.492242898	Yes	
PARK2	E3 ubiquitin-protein ligase parkin	2.482088122		
RELA	Transcription factor p65	2.468415262	Yes	
MAPK1	Mitogen-activated protein kinase 1	2.421196549	Yes	
FBXO6	F-box only protein 6	2.408499574		Yes
SP1	Transcription factor Sp1	2.371253556	Yes	
SUMO3	Small ubiquitin-related modifier 3	2.359854421		
UBXN7	UBX domain-containing protein 7	2.313191016	Yes	
PPP2CA	Serine/threonine-protein phosphatase 2A catalytic subunit alpha isoform	2.306173702	Yes	
NOTCH1	Neurogenic locus notch homolog protein 1	−2.157288489	Yes	
CREBBP	CREB-binding protein	−2.177580496		
ARRB1	Beta-arrestin-1	−2.190098767	Yes	
MDM2	E3 ubiquitin-protein ligase Mdm2	−2.211981126		
TRIM27	Zinc finger protein RFP	−2.29366386	Yes	
TARDBP	TAR DNA-binding protein 43	−2.294490786	Yes	
FLNA	Filamin-A	−2.303019202	Yes	
HDAC5	Histone deacetylase 5	−2.32463426		
PPP2R1A	Serine/threonine-protein phosphatase 2A 65 kDa regulatory subunit A alpha isoform	−2.340105915	Yes	
CTNNB1	Catenin beta-1	−2.39851723	Yes	
KDM1A	Lysine-specific histone demethylase 1A	−2.411266474	Yes	
HDAC1	Histone deacetylase 1	−2.469437565	Yes	
TGFBR1	TGF-beta receptor type-1	−2.471648327	Yes	
CBL	E3 ubiquitin-protein ligase CBL	−2.502979222	Yes	
TRAF2	TNF receptor-associated factor 2	−2.610641391		Yes
VHL	von Hippel-Lindau disease tumor suppressor	−2.722710155	Yes	
ATF2	Cyclic AMP-dependent transcription factor ATF-2	−2.723779846	Yes	
MYC	Myc proto-oncogene protein	−2.826430018	Yes	
APP	Amyloid-beta A4 protein	−3.151878516		Yes
SUMO1	Small ubiquitin-related modifier 1	−3.553233037	Yes	

Among the top 40 high impact genes we found polyubiquitin-C (UBC), epidermal growth factor receptor (EGFR), cullin-3 (CUL3), neddylin (NEDD8), obscurin-like protein 1 (OBSL1), F-box only protein 6 (FBXO6), TNF receptor-associated factor 2 (TRAF2), amyloid-beta A4 protein (APP) plays important regulatory roles in the infected DCs (Table 1). Extending this list (Supplementary Figure S2), we observed 14-3-3 protein epsilon (YWHAE) as an interesting novel regulator that also interacts with high impact gene 14-3-3 protein zeta/delta (YWHAZ). YWHAE was recently mentioned as interesting protein that interacts with an uncharacterized fungal protein AFUA_2G17170 (Remmele et al., 2015) the ortholog for which in *Candida albicans* is a virulence related protein (Soong et al., 2000). Moreover, the co-immunoprecipitation of YWHAE with MHC II in B-cell exosomes (Buschow et al., 2010) indicates its function is relevant to the immune response. Figure 7B depicts how the subnetworks of network router YWHAE significantly impacts cell behavior specific to the 6 h-mark of fungal infection. Although we describe YWHAE as an example case, other routers of reader's interest can be analyzed further based on the provided Supplementary Dataset 2. We provided the subnetworks .gml files for both the control and confronted subnetwork of the router and target genes that can be opened in cytoscape software (Shannon et al., 2003).

### Validation of the Metabolic Flux Predictions for *A. fumigatus*

**Figure 8:**
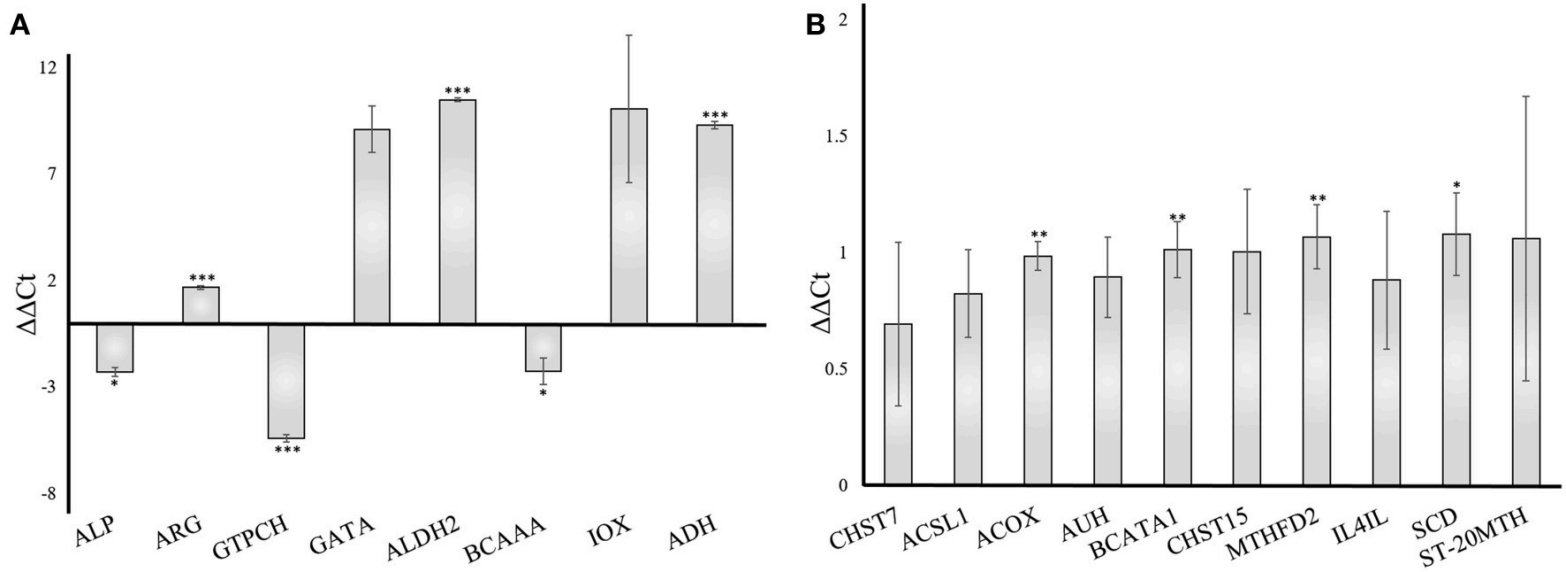
Validation of gene expression by qRT-PCR of *A. fumigatus* and human dendritic cells genes 6 h after infection. The data were normalized to and internal 18S RNA (*A. fumigatus*) and GADPH (human) control. Results are separated into *A. fumigatus* **(A)** and dendritic cell **(B)** gene expression. The error bars indicate the standard deviation (*n* = 3). ***p < 0.001, **p ≤ 0.01, *p ≤ 0.05. ALP, Alkaline phosphatase; ARG, Arginase; GTPCH, GTP cyclohydrolase; GATA, 4-aminobutyrate transaminase; ALDH2, Aldehyde dehydrogenase2; BCAAA, Branched-chain amino acid aminotransferase; IOX, Inositol oxygenase; ADH, Alcohol dehydrogenase; CHST7, carbohydrate sulfotransferase 7; ACSL1, acyl-CoA synthetase long-chain family member 1; ACOX, acyl-CoA oxidase; AUH, AU RNA binding methylglutaconyl-CoA hydratase; BCATA1, branched chain amino acid transaminase 1; CHST15, carbohydrate sulfotransferase 15; MTHFD2, methylenetetrahydrofolate dehydrogenase; IL4IL, interleukin 4 induced 1; SCD, stearoyl-CoA desaturase; ST-20MTH, ST20-MTHFS readthrough.

To validate the gene responses in the *A. fumigatus*-human interaction, we investigated an early stage of the confrontation (6 h) using qRT-PCR. The results from qRT-PCR revealed a considerable response of *A. fumigatus* genes, however, the human host responded more moderately. The *A. fumigatus* infection of DCs induced upregulated expression of 4-aminobutyrate transaminase GatA (AFUA_5G06680- GATA) and alcohol dehydrogenases (AFUA_5G06240 - ALDH2, AAA32684.1 - ADH). The same experiment revealed a decrease of alkaline phosphatase (AFUA_6G08710 – ALP), branched-chain amino acid aminotransferase (AFUA_1G01680 – BCAAA), and GTP cyclohydrolase I (AFUA_5G03140 - GTPCH) (Figure 8A), which are consistent with the bioinformatical analysis. The host response changes in gene expression were modest compared to the *A. fumigatus* gene expression. The timing of the PCR experiments was performed according to different previous papers after 6 h (Morton et al., 2011; Czakai et al., 2016). With later time points (gene expression data include even 9 h) you run into biological problems: there are no longer only conidia, but they germinate, and hyphae are generated, also some cells die. Hence, for validation we choose the somewhat earlier, but more secure time point where there are only conidia present.

We included 10 enzymes in our test and our analysis revealed a moderate increase of each examined enzyme (Figure 8B), in accordance to our pathway modeling results.

Our original hypotheses at the start of the study are hence replaced by a more complex and detailed picture of pathway changes: *A. fumigatus* is surprisingly well adapted to the challenge of stress due to DCs: most redox responses are quite, but catalase and alcohol dehydrogenase is active. Moreover, the differential activity of enzymes PEP carboxykinase, pdcA, LDHA, and aldehyde reductase *A. fumigatus* survive well in the anaerobic and nutrient deficient conditions in dendritic cell. Overall the metabolism is remarkable stable and well-adapted to environmental challenges, this could be a general feature *of A. fumigatus* a world-wide distributed and flourishing saprophyte. In contrast, the DCs reveal a number of specific features as fatty acid synthesis crucial for activating toll like receptor, glycosaminoglycan biosynthesis forming proteoglycans which can modulate immunological functions and active NOD like and RIG-I like receptor. Among the top 40 high impact genes we found polyubiquitin-C (UBC), epidermal growth factor receptor (EGFR), cullin-3 (CUL3), neddylin (NEDD8), obscurin-like protein 1 (OBSL1), F-box only protein 6 (FBXO6), TNF receptor-associated factor 2 (TRAF2), amyloid-beta A4 protein (APP) plays important regulatory roles in the infected DCs (Table 1). Extending this list (Supplementary Figure S2), we observed YWHAE as an interesting novel regulator that also interacts with high impact gene YWHAZ.

## Conclusions

The present study delivers a curated model of *A. fumigatus* metabolism as well as key subnetworks and involved flux modes. It looks in detail at the complex metabolic interplay and metabolic regulation between human DCs and *A. fumigatus*. Resting *A. fumigatus* conidia are metabolic inactive and inert. They can survive in this status for months or even years. Upon nutrition supply and optimal environmental factors, such as temperatures of above 30°C and humidity, conidia start to swell and soon germinate into germ tubes. Thus, in this condition, central metabolic pathways such as amino acid synthesis, fatty acid synthesis, and glycolysis are induced and mandatory for germination and subsequent fungal growth. Furthermore, *A. fumigatus* has established several mechanisms to circumvent immune surveillance and different virulence factors have been identified. Among them, catalases are important to detoxify oxidative threats and *A. fumigatus* catalase deletion mutants are more sensitive to H_2_O_2_. Gene expression data, EFMs and flux activity calculation together show potential pathways, usage of specific pathways and networks as well as flux strength of each pathway. From the pathogen side we identified eight pathways (ascorbate, beta-alanine, fatty acid, glycolysis, folate, arginine and proline, tryptophane, valin, leucine, and isoleucine metabolism) of key importance during DCs challenge and maintaining full growth potential in *A.fumigatus*. Enzymes arginase, inositol oxygenase, GTP cyclohydrolase, catalase, and others were found to be most active during dendritic cell challenge. Flux activity calculations from our model also suggest the important role of glucuronic acid for immune activation and host cell adaptation.

DCs rapidly get metabolically ready to sense and fight fungal cells, utilizing enzymes such as PPARα/PPARγ/PPARδ involved in lipid metabolism for getting their receptors and defenses ready. Besides well-known responders in DCs such as maturation markers CD80, CD83, and CD86 and interleukins, novel regulators in human DCs as identified by differential co-expression of genes in DCs during *A. fumigatus* confrontation include UBC, EGFR, and CUL3 with involved defense signaling networks.

## Materials and Methods

### Gene Expression Data and Pathway Enrichment

Gene expression data (GSE22053) (Morton et al., 2011) were analyzed from Gene Expression Omnibus (GEO) database (Barrett et al., 2011). DEGs relied on Significance Analysis of Microarrays using the MEV program (Chu et al., 2008) from the TM4 microarray data analysis suite (Saeed et al., 2006). In particular, we focused on the genes involved in fungal metabolism. Furthermore, metabolic pathway enrichment analysis of DEGs was performed (Huang da et al., 2009). Similarly, the pathways affected in human DCs during fungal infection were analyzed. The gene expression data (GSE69723) identified DEGs by comparing the expression of genes in human DCs with/without *A. fumigatus* challenge (Czakai et al., 2016).

We used the DAVID tool (Huang da et al., 2009) for the *A. fumigatus* data to accurately calculate whether a certain pathway or ontological classification of a pathway occurred more often in the DEGs then to be expected looking at all genes. The EASE Score [a modified Fisher Exact *p*-Value; (Hosack et al., 2003)] threshold 0.1 was used to identify the significantly over-represented pathways in both *A. fumigatus* and dendritic cell. Fisher's exact test was used to calculate the statistical significance of the over-represented pathway, followed by a correction for multiple testing to estimate the proportion of enriched gene sets that would occur by chance given the number of tested gene sets.

### Metabolic Networks and EFM Analysis

EFMs can be defined as “*a unique, minimal set of enzymes (participating reactions) to support steady state operation of a metabolic network with irreversible reactions to proceed in appropriate directions*” (Schuster et al., 1999, 2000, 2002). However, the computation of EFMs in genome-wide models of metabolism has to deal with the high number of possible modes (“combinatorial explosion”). To handle this, we applied the pathway-centric decomposition of *A. fumigatus* metabolic network considering each metabolic map individually (Schwartz et al., 2007) (Supplementary Figure S3). Orthologous reactions from metabolic models of *A. niger* (Andersen et al., 2008), *A. oryzae* (David et al., 2008), and *Aspergillus flavus* (Vongsangnak et al., 2008) were added to fill in the gaps for each pathway. This draft reconstruction was subsequently subjected to an iterative manual curation using sequence and domain analysis methods. Our approach further uses the integration of enrichment analysis with EFMs to elucidate the metabolic behavior of *A. fumigatus* in different conditions during host cell interference. Furthermore, our previously established *A. fumigatus* metabolic network (Kaltdorf et al., 2016) was used to elucidate the metabolic pathways by mapping of gene expression data. Multiple rules were adopted to define external metabolites in reaction network:

- a metabolite participating in many reactions was considered as buffered as its level will hardly be affected by the velocity of one particular reaction (Dandekar et al., 2003);
- branch points and metabolites that can only be either produced or consumed were considered as external (Dandekar et al., 2003);
- unbalanced ubiquitous metabolites were considered as external to avoid the existence of inactive metabolic branches.

The stoichiometric matrix was constructed for each pathway and EFMs were computed using a classical algorithm (Pfeiffer et al., 1999; Schwarz et al., 2007). The KEGG database (Kanehisa and Goto, 2000) and our previous model (Kaltdorf et al., 2016) was used to link annotated genes to protein and to reactions. A detailed Boolean gene-to-reaction mapping was employed to identify the expression state for each reaction. The calculated EFMs were classified as differential EFMs which define the significantly enriched EFMs with over-expressed reactions and involving a small number of under-expressed reactions based on differential expression during infection as defined in Rezola et al. (2014).

### Calculation of Pathway Activities

To identify actual flux strengths for each elementary mode we used YANAsquare. The gene expression data of *A. fumigatus* infecting the DCs at 3, 6, 9, and 12 h after infection was taken to model the flux activity. The gene expression data were fitted to the enzymes in the metabolic network model and we calculated the activities of all the enzymes in the model. The calculation error of fitting the expression data to enzyme activity has been estimated to be only a few percent (Cecil et al., 2011, 2015; Zirkel et al., 2012). YanaSquare uses an evolutionary algorithm (EA) to calculate flux activities using estimates of flux strength either from direct flux measurements (almost never available) or estimates from protein or gene expression data. The observed enzyme activity estimate is compared to the calculated enzyme activity summing up over all elementary modes the enzyme participates in and their calculated activity. This error is calculated for all enzymes of the network and then the pathway estimates are systematically varied by the evolutionary algorithm to minimize the total error (root mean square deviation; higher errors count more) between observed enzyme flux and calculated flux distribution (Schwarz et al., 2005). Here we used the gene expression data collected from *A. fumigatus* at different time points (0, 3, 6, 9, 12 h) of infection in DCs. A least square fit method used YANAsquare and next an R routine to calculate optimal pathway fluxes matching optimally the constraints for key enzyme activities. The latter were estimated according to significant elevated or lowered absolute gene expression intensity (Czakai et al., 2016).

### RNA Isolation and Quantitative RT-PCR

The total RNA was isolated separately from DCs from human, *A. fumigatus* cell culture and the infected human cell. For each sample cDNA was produced by reverse transcription from 1 μg of total RNA using the RevertAid First Strand cDNA Synthesis Kit (Fermentas). Resulting cDNA was diluted to a final concentration of 40 ng/μl. Gene expression was analyzed by qRT-PCR. The primers for selected *A. fumigatus* and human genes were designed by Geneious 9.0.5 (Kearse et al., 2012) with the estimated product size 150–200 bp with Tm values for each primer pair approximately 61°C (Supplementary Table S7). The experiment was performed in the StepOnePlus™ Real-Time PCR System (Applied Biosystems, Life Technologies GmbH, Darmstadt, Germany) in a 20 μl final volume containing 2x Absolute™ PerfeCTa™ SYBR™ Green FastMix™ (Rox) (Quanta Biosciences, Gaithersburg, MD, USA), 0.5 μM of each primer and 40 ng of cDNA sample. The reaction consisted of initial denaturation at 95°C for 5 min, followed by amplification including 40 cycles of 4 steps: denaturation at 94°C for 30 s, annealing at 60°C for 30 s, extension at 72°C for 30 s, and final extension at 72°C for 15 min was followed by melting curve analysis. Each assay was performed in triplicate and the negative control without cDNA template was included. Experiments were performed in triplicates and relative transcription level was normalized (ΔΔCt) to the housekeeping genes GADPH (human genes) and 18S RNA (*A. fumigatus* genes) as reference genes. Results were expressed as mean ± standard deviation (SD) and the data were evaluated using the GraphPad Prism 3.00 software by one sample *t*-test.

### Identification of Key Regulators in DCs

The context-based network modeling tool NetDecoder (da Rocha et al., 2016) was used to identify novel key regulators during dendritic cell confrontation to *A. fumigatus* from gene expression data (Figure 1). DEGs (GSE69723) were defined as source and transcriptional regulators were defined as sink that drive DCs battle. The impact score (IP) (verbatim definitions from da Rocha et al. (2016)] “*ranked genes based on their importance in mediating differences in information flow profiles between two given phenotypes. The impact score for gene gi is defined as*

$$\begin{array}{l} IP_{gi}\;=\;\left(giTF_{phenotype2}\;-\;giTF_{phenotype1}\right)\;\times \;NII\;\times \;NCD \end{array}$$

where giTF_phenotype2_ *is the total flow on gi in the phenotype 2 subnetwork, giTF*_*phenotype*1_ *is the total flow of gi in phenotype 1 subnetwork and NII is the number of new inflow interactions established by gi in the phenotype 2 subnetwork normalized by the sum of the total number of interactions established by gi in phenotype 1 and phenotype 2 subnetworks. NCD is the number of gene expression correlations that changed directionality (i.e., from positive correlation to negative correlation or vice versa) between phenotype 1 and phenotype 2, which is independent of the magnitude of gene expression correlations with the same directionality*.”

## Author Contributions

MS and SG performed the bioinformatics analyses. EB and EW carried out all validation experiments. EB performed the statistical analysis. MS and TD were involved in drafting the manuscript. JL and TD conceived the project and supervised the work. All authors were involved in manuscript writing and agreed to the final version of the manuscript.

### Conflict of Interest Statement

The authors declare that the research was conducted in the absence of any commercial or financial relationships that could be construed as a potential conflict of interest.
