# Supplementary material for: Aspergillus fumigatus Challenged by Human Dendritic Cells: Metabolic and Regulatory Pathway Responses Testify a Tight Battle
Source: Front Cell Infect Microbiol. 2019 May 22;9:168. doi: 10.3389/fcimb.2019.00168 (PMC6540932; doi:10.3389/fcimb.2019.00168)
Supplement: Supplementary file 4 [file Table_4.DOCX]

Supplementary Table S4: *Aspergillus fumigatus* enzymes and their metabolic response against dendritic cells

| Enzyme | Biological function | Reference |
| --- | --- | --- |
| Alcohol dehydrogenase | Adaptation to hypoxia | Grahl et al. 2011 (PMID: 21811407) |
| Alkaline phosphatase | Phosphate acquisition to promote fungal stress resistance to pH, cationic and oxidative stresses | Ikeh et al. 2017 (PMID: 28829379) |
| Arginase | Protection from reactive nitrogen species | Das et al. 2010 (PMID: 20585552), Wagener et al. 2017 (PMID: 28119468) |
| Branched chain amino acid transaminase | Response to amino acid starvation | Yin et al. 2004 (PMID: 15274137) |
| 4-aminobutyrate transaminase | GABA shunt known to accumulate during hypoxia | Wu et al. 2006 (PMID: 16896530) |
| GTP cyclohydrolase | Flavin synthesis regulating NO and ROS production | Asai et al. 2010 (PMID: 20230506) |
| Inositol oxygenase | Cell wall synthesis and energy production | Kanter et al. 2003 (PMID: 14663824) |
| Catalase | Protection from reactive oxygen species | Pradhan et al. 2017 (PMID: 28542620) |
