# Supplementary material for: Aspergillus fumigatus Challenged by Human Dendritic Cells: Metabolic and Regulatory Pathway Responses Testify a Tight Battle
Source: Front Cell Infect Microbiol. 2019 May 22;9:168. doi: 10.3389/fcimb.2019.00168 (PMC6540932; doi:10.3389/fcimb.2019.00168)
Supplement: Supplementary file 7 [file Table_7.DOCX]

Supplementary Table S7: The details of the primer pair used for the enzymes to be tested by the reverse transcriptase PCR experiments

*Aspergillus fumigatus* enzymes

| Enzyme name | Genbank ID | Primer |
| --- | --- | --- |
| 3-beta hydroxysteroid dehydrogenase/isomerase | AFUA_2G17850 | 3BHSD F: AGGAAGCCGCCAAAGTCAGT  3BHSD R: TGACTTCACAGGCCCAGACG |
| alcohol degydrogenase | AAA32684.1 | ADH F: CGTACCTGAAAGCACCTGTGT  ADH R: AATTGACTTCTTCGCCAGGGG |
| alcohol dehydrogenase | AFUA_5G06240 | ALDH2 F: ATGCCTCCAAAATCCCCAACG  ALDH2 R: CTGAAGGAATCTGGCGCTCG |
| inositol oxygenase | AFUA_2G10230 | IOX F: AAGGCATTCAACCCCTACGA  IOX R: TCCCAAAGAAGGTCCTCAAGT |
| GTP cyclohydrolase I | AFUA_5G03140 | GTPCH F: ACTTTGACGGGCTGAGTTGG  GTPCH R: TGCATTGGAGAGGACCCAGA |
| 4-aminobutyrate transaminase GatA | AFUA_5G06680 | GATA F: ACCTTCATCGCCTGGGACA  GATA R: TCCTGGAGAGCGTCGAGAAG |
| branched-chain amino acid aminotransferase | AFUA_1G01680 | BCAAA F: GGTCCAACGTCGGCTTCAA  BCAAA R: GGGGAGGAGACAAATTTGGGG |
| arginase | AFUA_3G11430 | ARG F: TCCTGTCCAAGCCAAATGAGC  ARG R: TTGAATCAAGTGCGCGACGA |
| aldehyde_reductase | AFUA_1G09750 | ALR F: GGATACCGCCACATTGACACG  ALR R: GGAACAATAAGCACCACCCCG |
| alkaline phosphatase | AFUA_6G08710 | ALP F: CCCACTGGCGCATTATTGGT  ALP R: TTGCGGGTGATTCTCATGCC |
| aldehyde dehydrogenase | AFUA_2G00720 | ALDH F: CGGCCATTGTGCCATGGAAT  ALDH R: CCTCCGAAAACTCCCCGCTA |
| spore-specific catalase CatA | AFUA_6G03890 | CATA F: AGATGCCGTCAAGTTCCCAGA  CATA R: GTACTTGCACCCAGAAGCCAC |
| 18S RNA | AB008401 | 18S RNA F: GGCCCTTAAATAGCCCGGT  18S RNA R: TGAGCCGATAGTCCCCCTAA |

Human enzymes

| Enzyme name | Genbank ID | Primer |
| --- | --- | --- |
| carbohydrate sulfotransferase 7 | NM_019886.3 | CHST7 F: GCACCCGGACGTTTTCTACTT  CHST7 R: CTCTTCCGCTGCGACTTCTC |
| Matrix metalloprotease 1 | CAA38691.1 | MMP1 F: GGCTGAAAGTGACTGGGAAACC  MMP1 R: TGCTCTTGGCAAATCTGGCGTG |
| phosphofructokinase | NM_001166686.1 | PFKM F: GCTGCCTACAACCTGGTGAAG  PFKM R: GGCTACGAAGTCCAGCTACCT |
| Human ligand activated transcription factor PPARgamma2 | U79012.1 | PPARgamma2 F: GACCCAGAAAGCGATTCCTTCA  PPARgamma2 R: GCTCCGTGGATCTCTCCGTAA |
| acyl-CoA dehydrogenase | NM_001608.3 | ACADSB F: AACCCGAGCATTTGTGGACAA  ACADSB R: AGGTTGGGGATACATGTGGGA |
| acyl-CoA synthetase long-chain family member 1 | NM_001995.3 | ACSL1 F: GCACTACTTGACAGCGACGAG  ACSL1 R: TAGGCTCTCGGAAACCAGACC |
| acyl-CoA oxidase 3 | NM_003501.2 | ACOX3 F: TTGCTAAGCTGTGTGTGCCAG  ACOX3 R: AAAAACTCGGGCAGAACGGTC |
| aldehyde dehydrogenase 9 family member A1 | NM_000696.3 | ALDH9A F: GGCTGGGACGTGCTTCATTAA  ALDH9A R: CTGTGTGTGTGGAGATGGGTG |
| AU RNA binding methylglutaconyl-CoA hydratase | NM_001698.2 | AUH F: CTCTGCAAAAATGGGCCTGGT  AUH R: AAGAAGCCAAAGCAGTGGGC |
| branched chain amino acid transaminase 1 | NM_005504.6 | BCAT1 F: GAGCCTGGAAAGGTGGAACTG  BCAT1 R: TAATGGGTGTCAGCAGGTCCT |
| carbohydrate sulfotransferase 15 | NM_015892.4 | CHST15 F: ACAGACCTCTATGACCGCCTG  CHST15 R: CCTAAGAGATGGGCTGCGAGA |
| 3-hydroxyacyl-CoA dehydratase 4 | NM_001010915.4 | HACD4 F: CCAGTTCTGTGGCCACTCTTG  HACD4 R: ATCTCTCCTGGAACTGCTGCA |
| hydroxyacyl-CoA dehydrogenase | NM_001184705.2 | HADH F: TTGTCCACAGCACAGACTTGG  HADH R: GCAACACTTCCTCCTTGCAGA |
| 4-aminobutyrate aminotransferase | NM_020686.5 | ABAT F: TGAGAGGACGAGGCACCTTT  ABAT R: TCAGGGATCACCACGCTCA |
| carbohydrate sulfotransferase 11 | NM_018413.5 | CHST14 F: GAATCTGCCGGATGGTGCTG  CHST14 R: TTTGGTGTGGACATCTGCTGC |
| carbohydrate sulfotransferase 14 | NM_130468.3 | CHST14 F: CACCACCTCACGTCCGATTTC  CHST14 R: CTCCCTCTTTGCCTACCCACT |
| Hexokinase 2 | NM_000189.4 | HK2 F: GAAGTGGGGTGGAGTGGAGAT  HK2 R: ACCACATTGTCCAGTGCATCG |
| methylenetetrahydrofolate dehydrogenase (NADP+ dependent) 2 | NM_006636.3 | MTHFD2 F: TCAAGGAAGGAGCAGCAGTCA  MTHFD2 R: GTGTTGGCCCCATGACAGTG |
| propionyl-CoA carboxylase alpha subunit | NM_000282.3 | PCCA F: GACAGTGGCATCCAACCAGG  PCCA R: AGGCACTGAAGAGAATGGCAG |
| phosphofructokinase | NM_001166686.1 | PFKM F: GCTGCCTACAACCTGGTGAAG  PFKM R: GGCTACGAAGTCCAGCTACCT |
| interleukin 4 induced 1 | NM_152899.1 | IL4I1 F: CAGAGGGTGATTGTGGTTGGC  IL4I1 R: AGGCAGATAACAGGATCGGGG |
| stearoyl-CoA desaturase | NM_005063.4 | SCD F: TCGTTGCCACTTTCTTGCGAT  SCD R: GGCTTCCACAACTACCACCAC |
| ST20-MTHFS readthrough | NM_001199760.1 | ST20-MTHFS F: CCGGTTCCAGAGCAATCACAT  ST20-MTHFS R: TCATCTTCATGCCAGGCCTTG |
| glyceraldehyde-3-phosphate dehydrogenase | NM_001289746.1 | GAPDH F: AGGGCTGCTTTTAACTCTGGT  GAPDH R: CCCCACTTGATTTTGGAGGGA |
