# Supplementary material for: Aspergillus fumigatus Challenged by Human Dendritic Cells: Metabolic and Regulatory Pathway Responses Testify a Tight Battle
Source: Front Cell Infect Microbiol. 2019 May 22;9:168. doi: 10.3389/fcimb.2019.00168 (PMC6540932; doi:10.3389/fcimb.2019.00168)
Supplement: Supplementary file 8 [file Table_8.DOCX]

Supplementary Figure1: Enzyme activities of all the enriched metabolic pathways in *Aspergillus fumigatus* during infection of dendritic cells

Pathway: Glycolysis/Gluconeogenesis

Pathway: Ascorbate and aldarate metabolic pathway

Pathway: Fatty acid degradation

Pathway: Valine leucine isoleucine degradation

Pathway: Arginine and Proline metabolism

Pathway: Tryptophan metabolism

Pathway: Beta-alanine metabolism

Pathway: Folate biosynthesis
