# Supplementary figures and images for: Aspergillus fumigatus Challenged by Human Dendritic Cells: Metabolic and Regulatory Pathway Responses Testify a Tight Battle
Source: Front Cell Infect Microbiol. 2019 May 22;9:168. doi: 10.3389/fcimb.2019.00168 (PMC6540932; doi:10.3389/fcimb.2019.00168)

**Supplementary Figure S2**


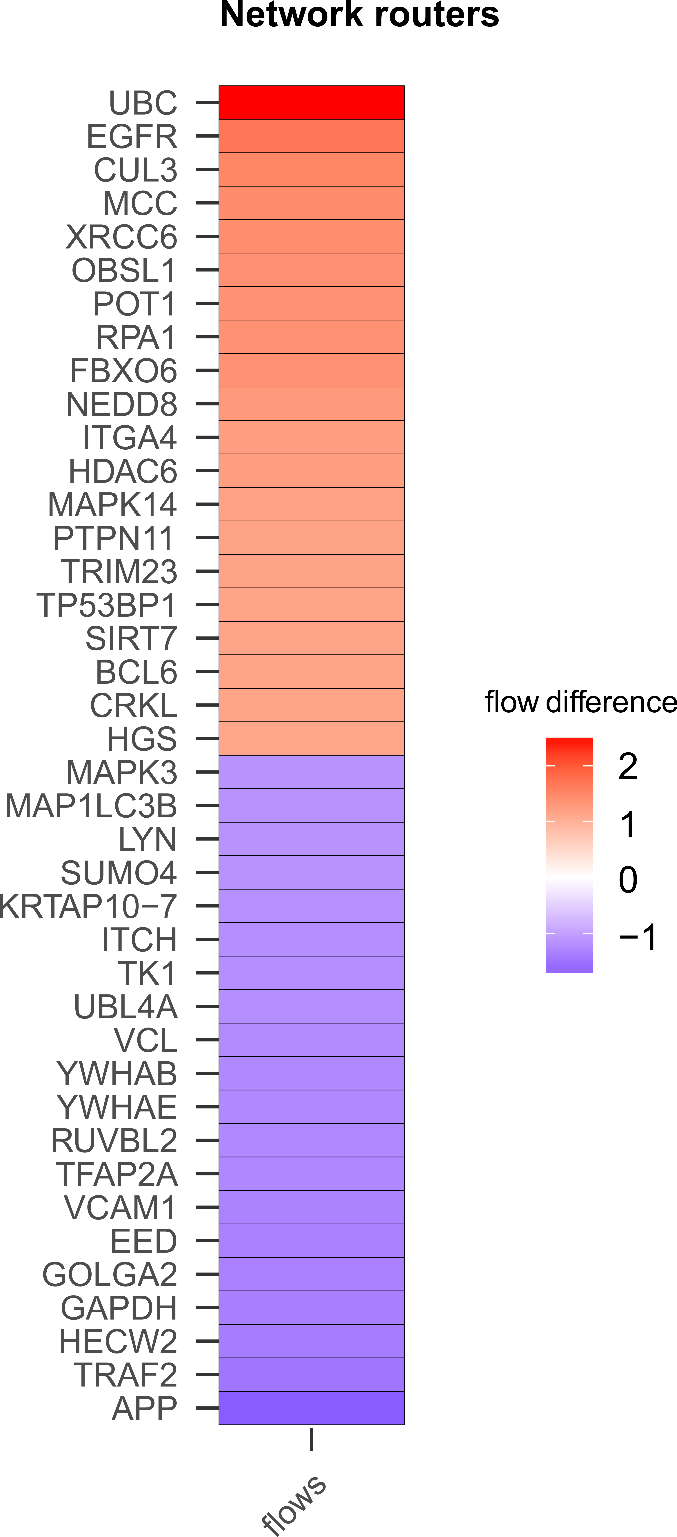

Supplement: Supplementary file 9 [file Table_9.DOCX]
