# Supplementary material for: Aspergillus fumigatus Challenged by Human Dendritic Cells: Metabolic and Regulatory Pathway Responses Testify a Tight Battle
Source: Front Cell Infect Microbiol. 2019 May 22;9:168. doi: 10.3389/fcimb.2019.00168 (PMC6540932; doi:10.3389/fcimb.2019.00168)
Supplement: Supplementary file 10 [file Table_10.DOCX]

Supplementary Figure S3: Analysis path of metabolic changes in Aspergillus fumigatus when challenged by a dendritic cell

Identification of subnetwork strongly affected by dendritic cell challenge using gene expression data

Mapping of gene expression data on elementary mode to understand which of these elementary modes are differentially expressed and hence active during the confrontation with a dendritic cell.

Decomposition of genome scale networks into sub networks considering metabolic map individually

Elementary mode analysis and flux activity calculation for individual enzymes by fitting the experimental expression data

Reconstruction of genome scale network

(Number of reactions, pathways)
